# Histone deacetylase inhibitor LMK235 attenuates vascular constriction and aortic remodelling in hypertension

**DOI:** 10.1111/jcmm.14188

**Published:** 2019-02-07

**Authors:** Sin Young Choi, Hae Jin Kee, Simei Sun, Young Mi Seok, Yuhee Ryu, Gwi Ran Kim, Seung‐Jung Kee, Marc Pflieger, Thomas Kurz, Matthias U. Kassack, Myung Ho Jeong

**Affiliations:** ^1^ Heart Research Center of Chonnam National University Hospital Gwangju Republic of Korea; ^2^ Hypertension Heart Failure Research Center Chonnam National University Hospital Gwangju Republic of Korea; ^3^ Molecular Medicine, Brain Korea 21 PLUS Chonnam National University Graduate School Gwangju Republic of Korea; ^4^ Zhoushan Hospital, Zhejiang University School of Medicine Zhoushan Zhejiang China; ^5^ National Development Institute of Korean Medicine Gyeongsan‐si Gyeongsangbuk‐do Republic of Korea; ^6^ Department of Laboratory Medicine Chonnam National University, Medical School and Hospital Gwangju Republic of Korea; ^7^ Institute of Pharmaceutical and Medicinal Chemistry Heinrich Heine University Düsseldorf Düsseldorf Germany

**Keywords:** calcium calmodulin‐dependent protein kinase II, HDAC5, hypertension, vascular hyperplasia, vasoconstriction

## Abstract

Here, we report that LMK235, a class I and histone deacetylase (HDAC6)‐preferential HDAC inhibitor, reduces hypertension via inhibition of vascular contraction and vessel hypertrophy. Angiotensin II‐infusion mice and spontaneously hypertensive rats (SHRs) were used to test the anti‐hypertensive effect of LMK235. Daily injection of LMK235 lowered angiotensin II‐induced systolic blood pressure (BP). A reduction in systolic BP in SHRs was observed on the second day when SHRs were treated with 3 mg/kg LMK235 every 3 days. However, LMK235 treatment did not affect angiotensin‐converting enzyme 1 and angiotensin II receptor mRNA expression in either hypertensive model. LMK235, acting via the nitric oxide pathway, facilitated the relaxing of vascular contractions induced by a thromboxane A2 agonist in the rat aortic and mesenteric artery ring test. In addition, LMK235 increased nitric oxide production in HUVECs and inhibited the increasing of aortic wall thickness in both animal hypertensive models. LMK235 decreased the enhanced cell cycle‐related genes cyclin D1 and E2F3 in angiotensin II‐infusion mice and restored the decreased p21 expression. In addition, LMK235 suppressed calcium calmodulin‐dependent protein kinase II (CaMKII) α, which is related to vascular smooth muscle cell proliferation. Inhibition or knockdown of HDAC5 blocked the CaMKIIα‐induced cell cycle gene expression. Immunoprecipitation demonstrated that class I HDACs were involved in the inhibition of CaMKII α‐induced HDAC4/5 by LMK235. We suggest that LMK235 should be further investigated for its use in the development of new therapeutic options to treat hypertension via reducing vascular hyperplasia or vasoconstriction.

## 1 INTRODUCTION

Hypertension may damage a variety of organs and leads to complications, such as brain stroke, retinopathy, atherosclerosis, kidney failure, coronary heart diseases and heart failure.^1^, ^2^, ^3^ Regulation of the arterial BP involves the renin‐angiotensin‐aldosterone system (RAAS). Angiotensin II, an oligopeptide, increases BP via vasoconstriction or sympathetic nerve stimulation. It also leads to thickening of the vascular wall, cell growth and cell migration.

Another class of protein involved in hypertension is histone deacetylases (HDACs). HDACs are enzymes that remove acetyl groups from histone and non‐histone proteins. HDACs are classified into four families: class I (HDAC1, 2, 3 and 8), class IIa (HDAC4, 5, 7 and 9), class IIb (HDAC6 and 10), III (sirtuin1‐7) and class IV (HDAC11). Enzyme activity of HDACs, as well as their expression, are reportedly associated with cardiac hypertrophy, hypertension, fibrosis, inflammation and cancer. For example, HDAC2 is positively implicated in cardiac hypertrophy via the increase in HDAC2 phosphorylation and its enzyme activity.^4^ HDAC1 and HDAC5 protein levels were increased in the lungs of human idiopathic pulmonary hypertension patients and hypoxia‐induced rats.^5^

There are several reports on the relevance of HDACs to the development of hypertension. Enhanced HDAC6 enzyme activity was shown in chronic hypertension such as deoxycorticosterone acetate (DOCA)‐salt‐induced hypertensive rats.^6^ Kidney damage is closely related to hypertension. HDAC7 plays an important role in the regulation of the epithelial Na (+) channel (ENaC) in the kidney via ubiquitination.^7^

HDAC3 plays a critical role in the regulation of hypertension, including in the deacetylation of mineralocorticoid receptor (MR) and its target gene transcriptional activity.^8^ HDAC4 interacts with both MR and HDAC3 and activates MR‐dependent transcription.^9^ Additionally, HDAC4 is involved in the control of hypertension via the inflammatory response.^10^ Recently, we demonstrated that HDAC4 expression or HDAC4 phosphorylation regulates arterial remodelling in angiotensin II‐induced hypertension.^11^ However, there is an unresolved issue that MC1568, a class II HDAC inhibitor, did not act selectively on HDAC4. Marek et al previously described LMK235 as a class IIa‐preferential hydroxamate‐based HDAC inhibitor.^12^

In the present study, we sought to investigate whether LMK235 could suppress high BP in angiotensin II‐infusion mice and in essential hypertensive rats. Our results suggest that LMK235 may be a useful pharmacological agent for the treatment of hypertension through relaxation of vascular contraction or attenuation of vascular hyperplasia although LMK235 does not act as a class IIa selective HDAC inhibitor.

## 2 MATERIALS AND METHODS

### 2.1 Hypertensive animal protocol

All animal procedures were approved by the Animal Experimental Committee of Chonnam National University Medical School (CNU IACUC‐H‐2014‐47) and carried out in accordance with the Guide for the Care and Use of Laboratory Animals (US National Institutes of Health Publications, 8th edition, 2011). CD‐1 mice (8 weeks old, 30‐35 g) were used in this study. Hypertension was induced by angiotensin II (Ang II, 1.3 mg/kg/day) infusion using 14‐day micro‐osmotic pumps (Alzet, model 1002; infusion rate of 0.25 μL/h) or saline alone. Ang II osmotic pumps were implanted subcutaneously after the injection of ketamine (120 mg/kg) and xylazine (6.2 mg/kg). One week after Ang II infusion, mice were administered LMK235 (1 or 3 mg/kg/day) or vehicle (dimethylsulphoxide [DMSO]) via intraperitoneal injection for 7 days. Mice were divided into three groups (n = 10‐12 per group): sham + vehicle, Ang II + vehicle, and Ang II + LMK235.

For the essential hypertension animal experiment, SHRs were used. The SHR is the most used model animal model for hypertension and is generally regarded as an essential hypertension model.^13^ SHRs (6 weeks old, approximately 170 g) were purchased from Charles River (Yokohama, Japan). Rats were intraperitoneally administered LMK235 or vehicle every 3 days for 2 weeks (n = 4 per group).

### 2.2 BP measurements

Systolic BP was measured by the tail‐cuff method (Visitech Systems, BP‐2000). Animals (mice and rats) were trained to allow for the measuring of BP before administration of the drug. Animals were trained to enter the holder at the same time more than three times a week to measure BP. For angiotensin II‐mice, systolic BP was measured when the mice were awake the day before they are killed. For SHRs, systolic BP was measured daily for 2 weeks.

### 2.3 Isometric tension measurement

Male Sprague‐Dawley rats were purchased from Orient Bio (Gyeonggi‐do, South Korea). The thoracic aortas and mesenteric arteries were excised and immersed in an ice‐cold, modified Krebs solution composed of NaCl (115 mmol/L), KCl (4.7 mmol/L), CaCl_2_ (2.5 mmol/L), MgCl_2_ (1.2 mmol/L), NaHCO_3_ (25 mmol/L), KH_2_PO_4_ (1.2 mmol/L) and dextrose (10 mmol/L). Mesenteric arteries are secondary branches of the main mesenteric trunk. The aortas and mesenteric arteries were cleaned by removing adherent connective tissue, soaked in the Krebs‐bicarbonate solution and cut into four ring segments (3.5 mm in length), as described previously.^14^ Some rings were denuded of endothelium by gently rubbing the internal surface with the edge of the forceps. Each aortic ring was suspended in a water‐jacketed organ bath (6 mL) maintained at 37°C and aerated with a mixture of 95% O_2_ and 5% CO_2_. Each ring was connected to an isometric force transducer (Danish Myo Technology, Skejbyparken, Aarhus N, Denmark). Rings were stretched to an optimal resting tension of 2.0 g, which was maintained throughout the experiment. Each ring was equilibrated in the organ bath solution for 90 minutes before the experiment involving the contractile response to 50 mmol/L of KCl addition. To determine the effect of LMK235 on the maintenance of vascular tension in rat endothelium‐intact or endothelium‐denuded aortic rings, vascular contractions were induced by thromboxane A2 agonist U46619 (30 nmol/L, 20 minutes). When each contraction reached a plateau, LMK235 was added cumulatively (0.1‐100 μmol/L) to elicit vascular relaxation.

### 2.4 Histology and immunohistochemistry

The paraffin‐embedded tissues were cut into 3‐μm‐thick sections, deparaffinized with xylene and then rehydrated with graded alcohol. Haematoxylin and eosin (H&E) staining was performed as described, with slight modifications.^15^ The arterial wall thickness was measured using NIS Elements Software (Nikon, Japan).

### 2.5 Cell culture

A10 vascular smooth muscle cells (VSMCs) were incubated with Dulbecco's modified Eagle medium (DMEM) with 4500 mg/L glucose containing 10% foetal bovine serum (FBS). Passages were carried out every 2 or 3 days. VSMCs were maintained in DMEM in the presence of 10% FBS with low glucose (1000 mg/L). Human umbilical vein endothelial cells (HUVECs) were obtained from Gibco (Waltham, MA, USA). HUVECs were grown in an endothelial cell basal medium (EBM) with EGM‐2 bullet kit (Lonza, Walkersville, MD) and maintained at 37°C under 5% CO_2_. Cells were cultured for 5‐7 passages.

### 2.6 Nitric oxide production in HUVECs

We used a nitric oxide‐sensitive fluorescence probe, 4‐amino‐5‐methylamino‐2′,7′‐difluorescein diacetate (DAF‐FM DA, Cayman), for nitric oxide detection. HUVECs were seeded on coverslips in 12‐well plates (1.5 × 10^5^/mL). Cells, serum‐starved with EBM for 16 hour, were treated with LMK235 (1 μmol/L) or _L_‐NAME (250 μmol/L) for 24 hour and incubated with DAF‐FM DA (2.5 μmol/L) for 45 minutes. Next, the cells were fixed with 70% ethanol for 45 minutes and mounted using Prolog Gold antifade reagent and DAPI (Invitrogen, USA).

### 2.7 MTT assay

Cell viability was tested by the MTT [3‐(4,5‐dimethylthiazol‐2‐yl)‐2,5‐diphenyl tetrazolium bromide] assay. A10 cells were seed into 24‐well culture dishes and serum‐starved overnight. Cells were treated with LMK235 or vehicle for 24 hours at the indicated concentrations. MTT reagent was added to the medium for 2 hours and then dissolved in DMSO, and the absorbance was measured at 570 nm. For assessing cell proliferation, A10 cells were transfected with pCMV‐SPORT6‐CaMKIIα in a dose‐dependent manner. The MTT assay was performed as described above.

### 2.8 Reagents

LMK235 was obtained from Prof. Thomas Kurz (Heinrich‐Heine Universität Düsseldorf, Germany) and synthesized according to a previously published protocol.^12^ HDAC5 small interfering RNA (siRNA) was purchased from Dharmacon (Lafayette, CO, USA). Sicontrol was purchased from Bioneer (Daejeon, South Korea). The *pCMV‐SPORT6‐CaMKII*α clone was provided from Korea Human Gene Bank, Medical Genomics Research Center, KRIBB, Korea.

### 2.9 HDAC enzyme activities

Enzyme activity measurements of class I and IIa HDACs were performed using a cell‐free system and according to a method previously described.^16^ We used fluorogenic HDAC1 (#50061), HDAC2 (#50062), HDAC3 (#50063), HDAC4 (#50064), HDAC5 (#50065), HDAC6 (#50076), HDAC7 (#50067), HDAC8 (#50068) and HDAC9 (#50069) enzyme assay kits from BPS Bioscience (San Diego, CA, USA). To test the enzyme activity of LMK235, 0.001, 0.003, 0.01, 0.03, 0.1, 0.3, 1, 3 and 10 μmol/L concentrations were used. For the IC_50_ calculations, every data point was normalized to the vehicle (100% activity). The normalized data were fitted with a Hill non‐linear curve fit (OrigionPro 9.0). Employing the “Find X from Y” function in OrigionPro 9.0 resulted in the IC_50_ values (50% activity).

### 2.10 Transfection

A10 cells were seeded into 12‐well dishes. A10 cells were transfected with the indicated concentrations of pCMV‐SPORT6‐CaMKIIα. After 24 hours of transfection, total RNA was extracted and quantitative real time polymerase chain reaction (qRT‐PCR) was performed. For knockdown of HDAC5, rat HDAC5 siRNA or sicontrol was used in A10 cells using RNAiMAX reagent according to the manufacturer's guidelines. A concentration of 100 nmol/L of siRNA was used.

### 2.11 Real‐time reverse transcription‐polymerase chain reaction

Total RNA from aortic tissue was isolated with TRIzol reagent (Invitrogen Life Technologies), and 1 μg of RNA was used for the reverse transcription reaction with TOPscript RT DryMIX (Enzynomics, South Korea). Quantification of mRNA levels was performed with the SYBR Green PCR kit (Enzynomics, South Korea). The PCR primers used in this study are shown in Table S1.

### 2.12 Western blot analysis

Cells were lysed in RIPA lysis buffer [150 mmol/L NaCl, 1% Triton X‐100, 1% sodium deoxycholate, 50 mmol/L Tris‐HCl pH 7.5, 2 mmol/L EDTA, 1 mmol/L PMSF, 1 mmol/L DTT, 1 mmol/L Na_3_VO_4_, and 5 mmol/L NaF] with protease inhibitor cocktail and sonicated. Approximately 30 μg of protein was separated by 10% sodium dodecyl sulphate‐polyacrylamide gel electrophoresis (SDS‐PAGE) and transferred to polyvinylidene difluoride (PVDF) membranes. After blocking with 5% skim milk for 1 hour, proteins were incubated with the primary antibodies overnight: anti‐cyclin D1 (1:1000), anti‐HDAC1 (1:1000), anti‐HDAC2 (1:1000), anti‐HDAC4 (1:1000) and anti‐HDAC5 (1:1000). As secondary antibodies, horse radish peroxidase‐linked antimouse or anti‐rabbit antibodies were used. Protein bands were visualized using Immobilon Western Detection Reagents (Millipore, Billerica, MA, USA). The Bio‐ID software was used to quantify protein expression (Vilber Lourmat, Eberhardzell, Germany).

### 2.13 Immunoprecipitation

In order to investigate the interaction between HDAC4/5/6 and class I HDACs, we performed immunoprecipitation in VSMCs. Protein was separated using 0.5% NP lysis buffer (50 mmol/L Tris, pH 8.0; 150 mmol/L NaCl; 1 mmol/L EDTA; 0.5% Nonidet P‐40 (Igepal); 1 mmol/L PMSF; protease inhibitors) in VSMC and incubated with indicated HDAC antibodies (HDAC1, HDAC2, HDAC3) or normal IgG antibody at 4°C overnight. Following incubation of protein A/G agarose beads (Santa Cruz) with HDAC‐enriched proteins for 1 hour at 4°C, the beads were washed thrice for 5 minutes with 0.5% NP buffer. Proteins bound to the beads were eluted into the sample buffer. Following SDS‐PAGE loading and transfer to a PVDF membrane, blots were incubated with HDAC4, HDAC5 or HDAC6 antibodies (Santa Cruz). For input determination, 25 μg of total protein were subjected to SDS‐PAGE. Immunoblotting was performed using anti‐HDAC1, anti‐HDAC2, anti‐HDAC3, anti‐HDAC4, anti‐HDAC5 and anti‐HDAC6.

### 2.14 Statistical analysis

All the data are expressed as the mean ± standard error (SE). Data analysis was performed using ANOVA with a Bonferroni post hoc test when three or more groups were compared. The statistics were analysed using GraphPad Prism version 5 and a *P* < 0.05 was considered significant.

## 3 RESULTS

### 3.1 LMK235 reduces high BP in angiotensin II‐induced hypertensive mice and SHRs

**Figure 1:**
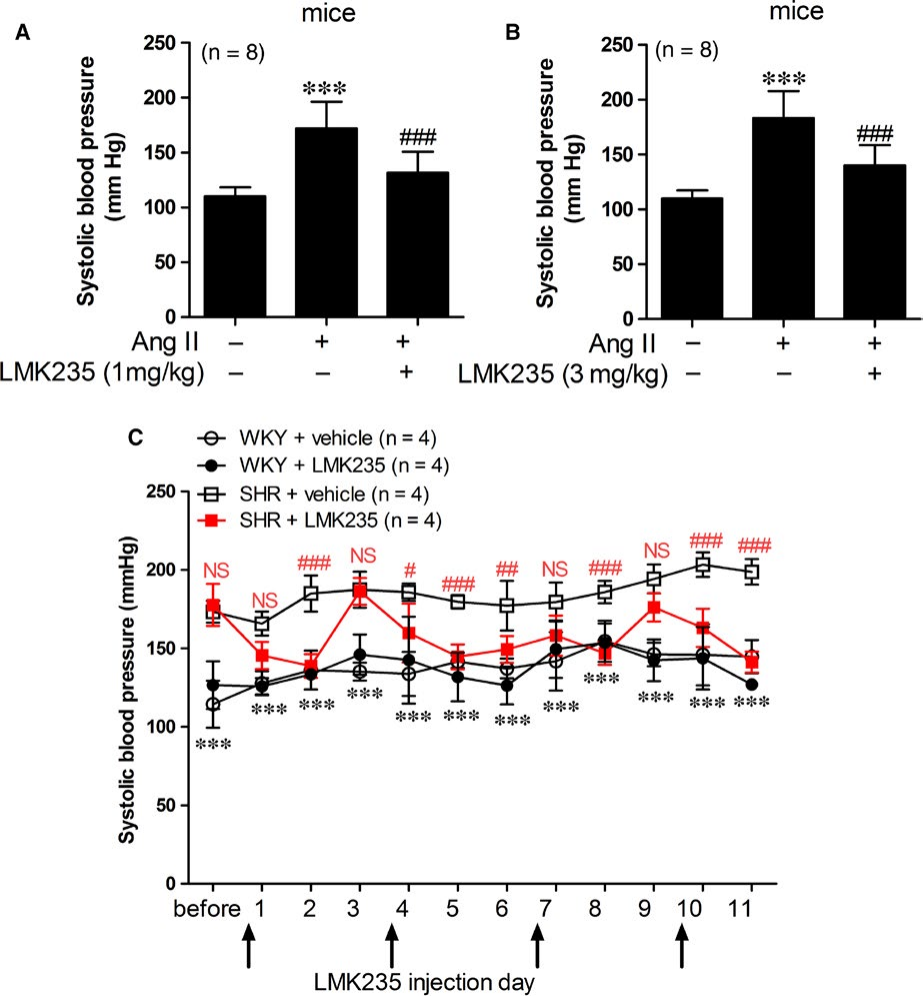
LMK235 reduces high blood pressure in angiotensin II‐induced hypertensive mice and spontaneously hypertensive rats (SHRs). (A‒B) Systolic blood pressures were measured in vehicle‐treated control, vehicle‐treated angiotensin II‐infusion (Ang II) and LMK235‐treated angiotensin II‐infusion (Ang II + LMK235) mice (n = 8 each). Administration of LMK235 was started 1 wk after angiotensin II‐infusion in mice. Mice were given LMK235 daily (A; 1 mg/kg/d, B; 3 mg/kg/d). ***P < 0.001 vs vehicle‐treated control; ^###^ *P* < 0.001 vs vehicle‐treated angiotensin II‐infusion mice. (C) Systolic blood pressures were determined in WKY control rats, LMK235‐treated WKY rats, vehicle‐treated SHRs and LMK235‐treated SHRs (n = 4 each). LMK235 was administered every 3 d, and systolic blood pressures were measured daily. ***P < 0.001 (vehicle‐treated WKY control vs vehicle‐treated SHRs, black); ^#^ *P* < 0.05, ^##^ *P* < 0.01 and ^###^ *P* < 0.001 (vehicle‐treated SHRs vs LMK235‐treated SHRs, red); NS indicates not significant

We recently demonstrated that MC1568 (class II HDAC inhibitor) but not tubastatin A (HDAC6 selective inhibitor) lowered elevated BP in angiotensin II‐induced hypertensive mice.^11^, ^17^ Therefore, we suggested that HDAC4 and HDAC5 may be related to the development of hypertension. We thus tested the effect of LMK235, a previously described HDAC4‐ and HDAC5‐selective inhibitor, at different dosages in two hypertension animal models. First, we examined the anti‐hypertensive effect of LMK235 in angiotensin II‐induced hypertension. We found that daily LMK235 administration (1 mg/kg/day) is sufficient to lower systolic BP in angiotensin II‐infusion mice (Figure 1A). In addition, a moderate dosage of 3 mg/kg/day LMK235 showed a similar effect on the inhibition of high BP (Figure 1B). Next, we investigated the BP lowering effect of LMK235 on SHRs. LMK235 was administrated to SHRs every 3 days, and systolic BP was measured daily. Before LMK235 treatment, SHRs showed high systolic BP compared to WKY controls. On the next day after LMK235 injection, a reduction in systolic BP relative to SHRs was observed. At 2 days after LMK235 treatment, systolic BP was significantly lowered in SHRs. At 3 days after LMK235 administration, systolic BP was restored to a comparable level to vehicle‐treated SHRs (Figure 1C). The cycle‐pattern of systolic BP repeatedly occurred. LMK235 showed a 2‐day long‐lasting BP lowering effect.

### 3.2 Expression of ACE1 and AT1 mRNA are not reduced by LMK235

**Figure 2:**
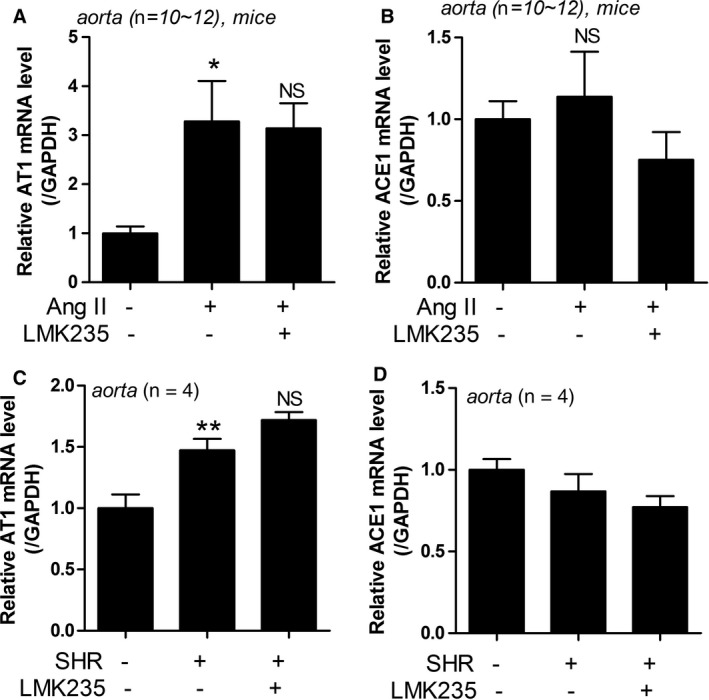
LMK235 did not reduce the expression of ACE1 and AT1 mRNA in hypertension. (A‐B) The expressions of AT1 and ACE1 mRNA levels were assessed by qRT‐PCR in aortas of angiotensin II‐infusion mice. *P < 0.05 vs control; NS indicates not significant. (C‐D) The expressions of AT1 and ACE1 mRNA levels were assessed by qRT‐PCR in aortas from SHRs. **P < 0.01 vs WKY control; NS indicates not significant

To determine whether LMK235 can regulate hypertension through the RAAS, we examined the mRNA levels of angiotensin II type 1 receptor (AT1) and angiotensin‐converting enzyme 1 (ACE1) in the aorta from angiotensin II‐infusion mice and SHRs. AT1 transcript levels were significantly increased in angiotensin II‐infusion mice. The increase was not reduced by LMK235 treatment (Figure 2A). ACE1 mRNA levels were not up‐regulated in angiotensin II‐infusion mice (Figure 2B). We observed that AT1 mRNA levels were significantly enhanced in the aortas of SHRs, but they were not affected by LMK235 administration (Figure 2C). ACE1 mRNA was not increased in the aortas of SHRs (Figure 2D).

### 3.3 LMK235 induces vasorelaxation via the nitric oxide pathway

**Figure 3:**
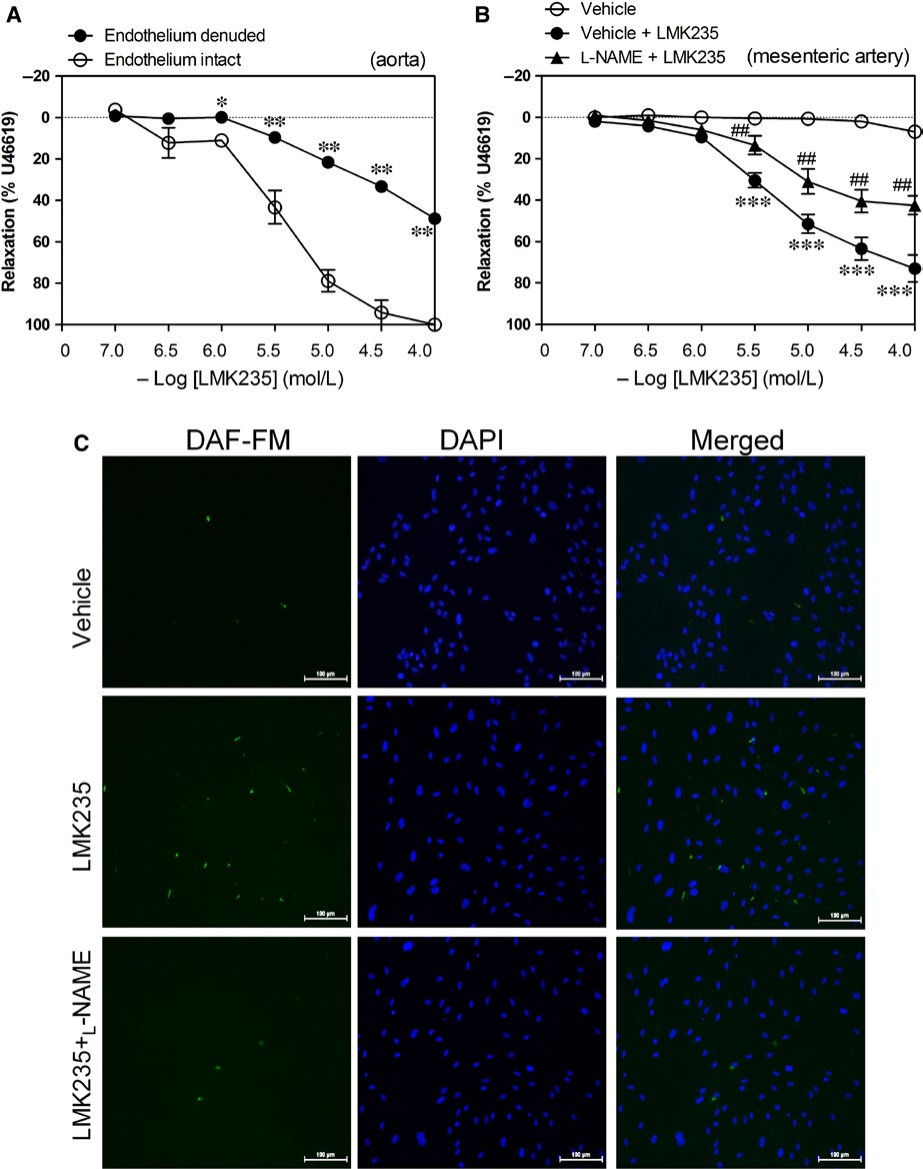
Pretreatment with _L_‐NAME reduces LMK235‐mediated vasorelaxation in rat mesenteric arteries as well as LMK235‐induced nitric oxide production in HUVECs. (A) LMK235 was added cumulatively to elicit relaxation when vascular contraction induced by U46619 (30 nmol/L) reached plateaus in endothelium‐intact (open circle) or endothelium‐denuded (black circle) rat aortic rings. Relaxation is expressed as a percentage of the maximal contraction. Data are expressed as mean ± SEM. *P < 0.05 and **P < 0.01 vs endothelium intact at the same concentration points of LMK235. (B) Mesenteric artery rings were pretreated with _L_‐NAME (100 μmol/L) or vehicle (0.1% dimethylsulphoxide) for 30 min. LMK235 or vehicle was added cumulatively to elicit relaxation when vascular contractions in rat mesenteric arteries induced by U46619 reached a plateau (n = 4). Data are expressed as mean ± SEM. ***P < 0.001 vs vehicle‐treated group; ^##^ *P* < 0.01 vs vehicle‐treated LMK235 group at the same concentration points of LMK235. (C) HUVECs were treated with LMK235 (1 μmol/L) in the presence or absence of _L_‐NAME (250 μmol/L) for 24 h and labelled with a fluorescent nitric oxide indicator, DAF‐FM DA (2.5 μmol/L), for 30 min. Three independent experiments were conducted, and representative images are shown. Scale bar = 100 μm

To elucidate whether the anti‐hypertensive effect of LMK235 is related to vascular contraction, we investigated vascular contraction‐relaxation in rat thoracic aortas and mesenteric arteries. LMK235 was added cumulatively (0.1‐100 μmol/L) to elicit relaxation after induction of vascular contraction by U46619 (30 nmol/L), a thromboxane A2 agonist, until reaching plateaus in endothelium‐intact or endothelium‐denuded rat aortic rings. As shown in Figure 3A (open circles), LMK235 treatment dose dependently reduced U46619‐induced vasoconstriction in endothelium‐intact aortic rings. In addition, LMK235 also inhibited U46619‐induced vasoconstriction in endothelium‐denuded aortic rings (Figure 3A, black circles). However, the relaxation effect of LMK235 was stronger in endothelium‐intact aortic rings than in endothelium‐denuded aortic rings. The vascular relaxation effect of LMK235 showed similar results in rat mesenteric arteries (Figure 3B). In order to determine whether LMK235 relaxes vascular contraction via activation of the nitric oxide pathway, we pretreated mesenteric artery rings with a nitric oxide synthase blocker, _L_‐NAME (100 μmol/L), or vehicle for 30 minutes. LMK235 was cumulatively added to U46619‐treated mesenteric vessels. Pretreatment with _L_‐NAME inhibited vascular relaxation, which was induced by cumulative addition of LMK235 (Figure 3B). To assess whether the rat ring test results could be replicated in vitro, we measured nitric oxide production in HUVECs. LMK235 increased nitric oxide production in HUVECs, and this increase was reduced by _L_‐NAME treatment (Figure 3C).

### 3.4 LMK235 decreases the increased aortic wall thickness in hypertension

**Figure 4:**
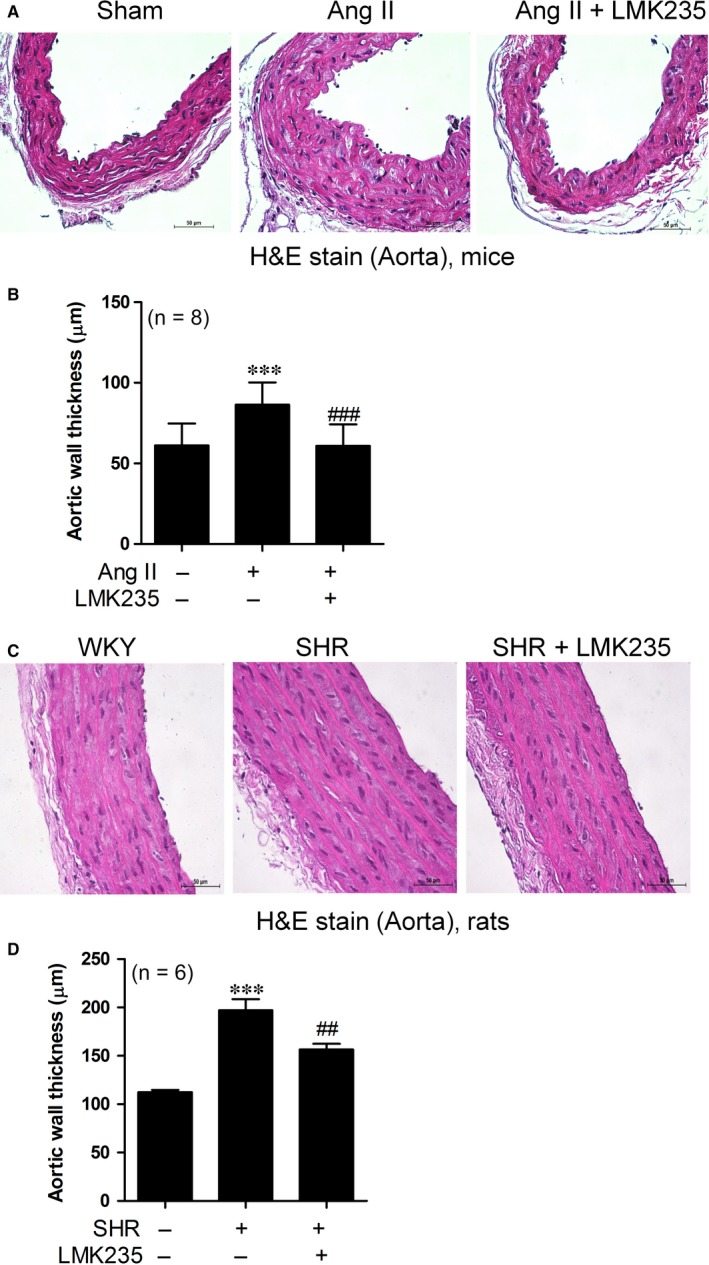
LMK235 decreases aortic wall thickness in angiotensin II‐induced hypertensive mice and spontaneously hypertensive rats (SHRs). (A) Representative images of haematoxylin and eosin (H&E)‐stained thoracic aortas (scale bar, 50 μm). Ang II indicates angiotensin II‐infusion mice. (B) Aortic wall thickness was quantified from H&E staining (n = 8 per group). ***P < 0.001 vs control; ^###^ *P* < 0.001 vs Ang II. (C) H&E staining in vehicle‐treated WKY, vehicle‐treated SHRs and LMK235‐treated SHR aortas (scale bar, 50 μm). (D) Quantification of aortic wall thickness (n = 6 per group). ***P < 0.001 vs vehicle‐treated WKY; ^##^ *P* < 0.01 vs vehicle‐treated SHRs

Previously, we demonstrated that MC1568 attenuates vascular hypertrophy and hyperplasia both in angiotensin II‐induced hypertensive mice and VSMCs.^11^ To determine whether LMK235 can affect arterial wall thickness, we measured wall thickness after H&E staining. As shown in Figure 4A,B, LMK235 treatment for 1 week with 3 mg/kg/day completely inhibited the increased aortic wall thickness in angiotensin II‐infusion mice. Similar results were observed in the SHR aortas (Figure 4C,D). Aortic wall thickness of SHRs was increased compared to WKY, and this increase was reduced by LMK235 treatment.

### 3.5 LMK235 decreases the expression of cell cycle genes and calcium CaMKII α in angiotensin II‐infusion mice

**Figure 5:**
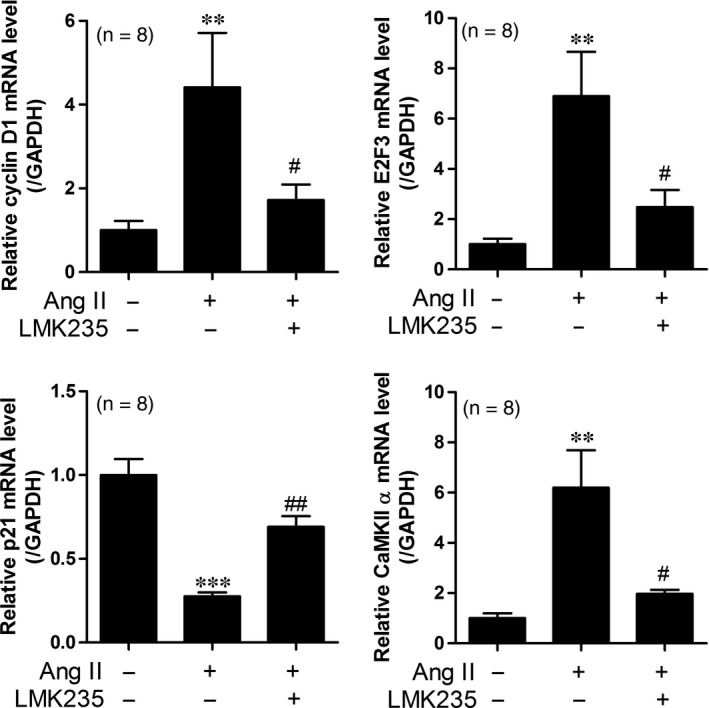
LMK235 decreases the expression of cell cycle genes and calcium calmodulin‐dependent protein kinase II (CaMKII) α in angiotensin II‐infusion mice. (A‐B) Transcript levels of cyclin D1 and E2F3 were determined by qRT‐PCR in the aortas from angiotensin II‐infusion mice (n = 8 per group). (C) The p21 mRNA levels were analysed by qRT‐PCR in the aortas from angiotensin II‐infusion mice. (D) CaMKIIα mRNA levels were assessed in the aortas from angiotensin II‐infusion mice. **P < 0.01 and ***P < 0.001 vs control; ^#^ *P* < 0.05 and ^##^ *P* < 0.01 vs angiotensin II‐infusion mice

To identify whether arterial wall thickness may be mediated by VSMC growth, we examined the cell cycle‐related molecules using qRT‐PCR. LMK235 administration significantly suppressed the expression of cyclin D1 and E2F3 mRNA levels (Figure 5A,B). Next, we determined the expression of p21, a cell cycle arrest gene. We found that p21 transcript levels were decreased in the aortas of angiotensin II‐infusion mice compared to the control. LMK235 restored the repressed p21 expression (Figure 5C). Calcium calmodulin‐dependent kinase II (CaMKII) has been reported to be related to the proliferation of VSMCs.^18^, ^19^ In addition, we have reported that the overexpression of the CaMKIIα isoform increases VSMC proliferation.^11^ Therefore, we determined the expression of CaMKIIα in angiotensin II‐infusion mice. As shown in Figure 5D, LMK235 significantly reduced the angiotensin II‐induced CaMKIIα mRNA levels.

### 3.6 LMK235 decreases CaMKIIα overexpression‐induced cell cycle genes and class I/IIa HDACs

**Figure 6:**
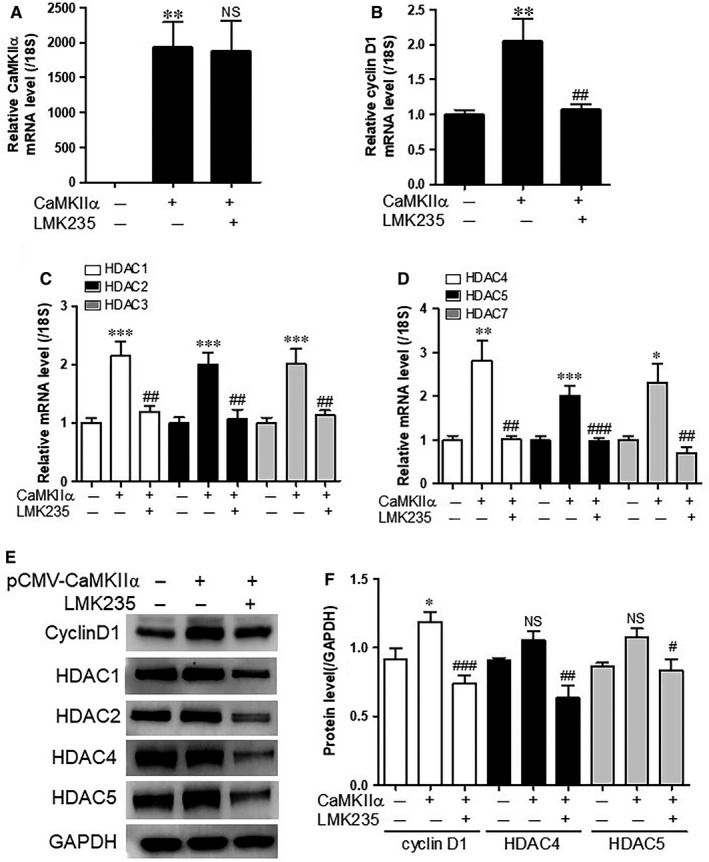
LMK235 decreases CaMKIIα overexpression‐induced cell cycle genes and class I/IIa HDACs. (A‐D) A10 cells were transfected with *pCMV‐SPORT6‐CaMKII*α or *pCMV‐SPORT6* empty vector and treated with LMK235 (1 μmol/L) for 24 h. Transcript levels of CaMKIIα, cyclin D1 and class I/IIa HDACs were determined by qRT‐PCR. Target genes were normalized to 18S rRNA. Data are expressed as mean ± SE of three independent experiments. *P < 0.05, **P < 0.01 and ***P < 0.001 vs empty vector; ^##^ *P* < 0.01 and ^###^ *P* < 0.001 vs CaMKIIα transfection group; NS indicates not significant. (E and F) Western blot analyses of cyclin D1, HDAC1, HDAC2, HDAC4 and HDAC5. GAPDH was used as a loading control. The amount of protein expression was quantified by densitometry. *P < 0.05 vs empty vector; ^#^ *P* < 0.05, ^##^ *P* < 0.01 and ^###^ *P* < 0.001 vs CaMKIIα transfection group

In the present study, we confirmed that overexpression of CaMKIIα increased A10 cell proliferation (Figure S1A,B). To further elucidate the relevance of CaMKIIα to VSMC proliferation, the effect of forced CaMKIIα expression on cell cycle‐related genes was investigated. The overexpression of CaMKIIα successfully increased CaMKIIα in A10 cells (Figure S2A). We observed that CaMKIIα transfection induced cyclin D1 mRNA levels (Figure S2B). To identify whether LMK235 can affect the cell cycle genes induced by overexpression of CaMKIIα, we first performed an MTT assay. We found that cell viability was not affected by LMK235 treatment up to 1000 nmol/L concentration (Figure S3). Treatment with LMK235 (1000 nmol/L) for 24 hours in A10 cells did not change the mRNA levels of overexpressed CaMKIIα (Figure 6A). LMK235 treatment reduced the mRNA levels of cyclin D1 induced by overexpression of CaMKIIα in A10 cells (Figure 6B). To investigate whether CaMKIIα directly affects the expression of class I/IIa HDACs, we transfected CaMKIIα into A10 cells in a concentration‐dependent manner. CaMKIIα forced expression increased mRNA expression of HDACs belonging to class I and class IIa, except for HDAC8 and HDAC9 (Figure S4A,B).

To determine whether LMK235 affects the expression of HDAC, qRT‐PCR and Western blotting were performed. Overexpression of CaMKIIα increased mRNA expression of class I HDACs (HDAC1, 2, 3) and class IIa HDACs (HDAC4, 5, 7) similarly (Figure 6C,D). In addition, LMK235 significantly reduced the mRNA expression of all six HDACs.

At the protein level, CaMKIIα‐induced cyclin D1 was reduced by LMK235 treatment (Figure 6E,F). Although HDAC4 and HDAC5 protein expression did not increase statistically as a result of overexpression of CaMKIIα, LMK235 treatment significantly reduced HDAC4 and HDAC5 proteins (Figure 6E,F). In addition, protein expression of HDAC1 and HDAC2 was significantly reduced by LMK235 treatment (Figures 6E and S5). The findings indicated that LMK235 reduced protein expression in class I and IIa/b HDAC following LMK235 treatment without CaMKIIα transfection to A10 cells (Figure S6A,B).

### 3.7 HDAC4 and 5 interact endogenously with class I HDACs in VSMCs

**Figure 7:**
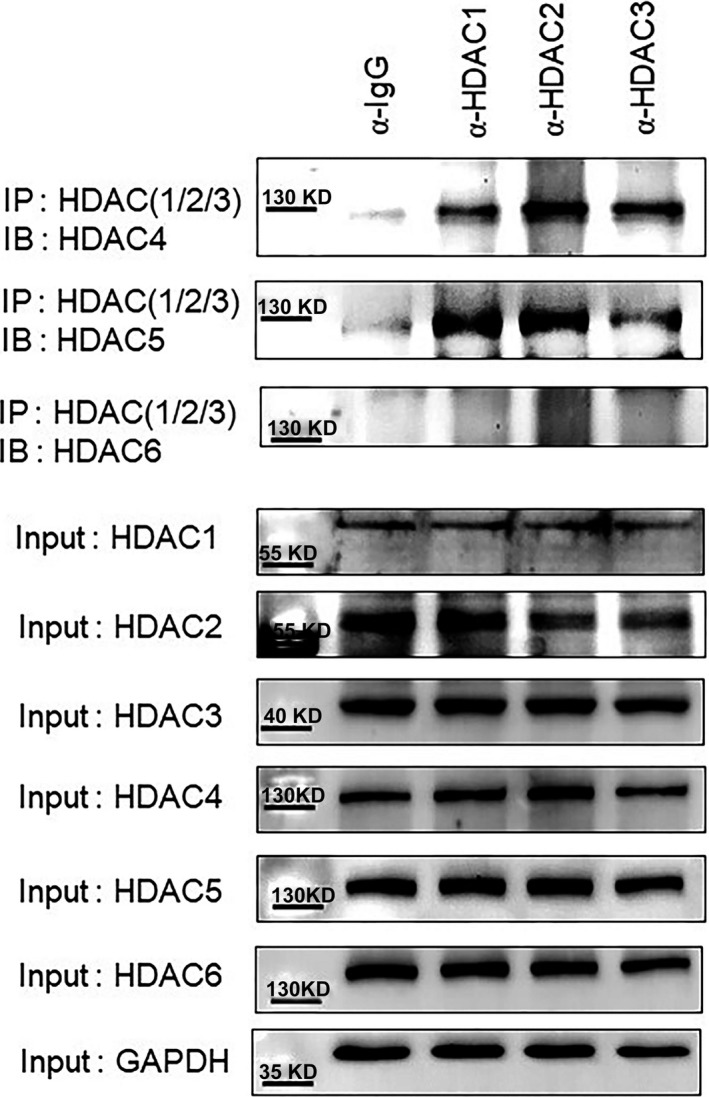
HDAC4 and 5 interact with class I HDACs in VSMCs. Protein lysates from VSMCs were immunoprecipitated with anti‐normal IgG or anti‐HDAC1, anti‐HDAC2 and anti‐HDAC3 antibodies followed by immunoblotting with anti‐HDAC4, anti‐HDAC5 and anti‐HDAC6 antibodies. The above three blots depict immunoprecipitation and lower seven blots depict input. IP; immunoprecipitation, IB; immunoblotting

To investigate whether inhibition of HDAC4/5 by LMK235 is caused by recruiting class I HDACs, we performed immunoprecipitation in VSMCs. HDAC4 interacted endogenously with HDAC1, HDAC2 and HDAC3 (Figure 7). HDAC5 was strongly associated with HDAC1 and HDAC2, but interacted weakly with HDAC3. However, HDAC6 did not bind to HDAC1, HDAC2 or HDAC3.

### 3.8 Knockdown of HDAC5 attenuates CaMKIIα overexpression‐induced cell cycle genes

**Figure 8:**
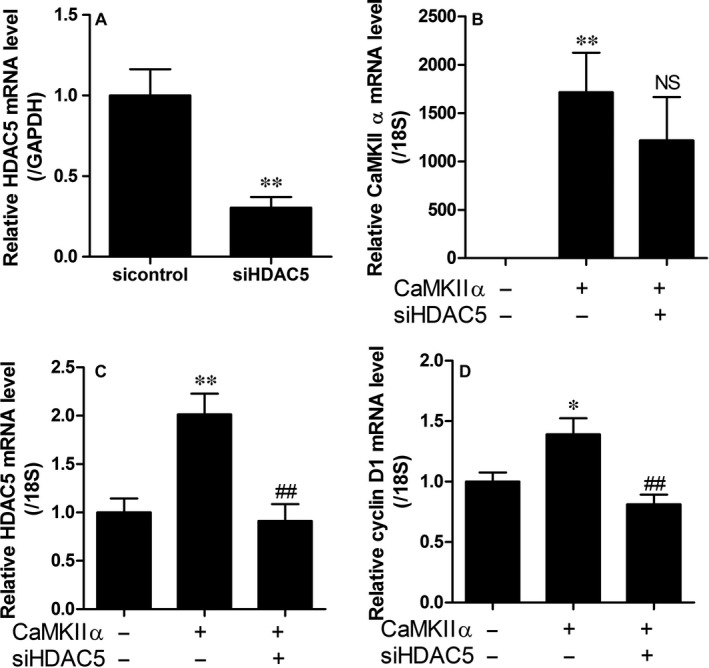
Knockdown of HDAC5 decreases CaMKIIα overexpression‐induced cell cycle genes. (A) A10 cells were transfected with HDAC5 siRNA or sicontrol. Transcript levels of HDAC5 were assessed by qRT‐PCR. **P < 0.01 vs sicontrol. (B‐D) A10 cells were transfected with *pCMV‐SPORT6‐CaMKII*α or *pCMV‐SPORT6* empty vector. Twenty‐four hours after transfection, cells were transfected with siHDAC5 or sicontrol for an additional 24 h. Transcript levels of CaMKIIα, HDAC5 and cyclin D1 were determined by qRT‐PCR. Target genes were normalized to GAPDH or 18S rRNA. Data are expressed as the mean ± SE of three independent experiments. *P < 0.05 and **P < 0.01 vs sicontrol; ^##^ *P* < 0.01 vs CaMKIIα transfection group

To examine whether HDAC5 can regulate CaMKIIα‐induced expression of cell cycle genes, knockdown of HDAC5 was performed. HDAC5 siRNA successfully reduced the endogenous HDAC5 transcript levels in A10 cells (Figure 8A). However, HDAC5 siRNA did not affect the overexpression of CaMKIIα (Figure 8B). Transfection of HDAC5 siRNA completely inhibited the mRNA levels of HDAC5 induced by the overexpression of CaMKIIα (Figure 8C). In addition, siHDAC5 decreased cyclin D1 mRNA levels induced by overexpression of CaMKIIα (Figure 7D).

## 4 DISCUSSION

We investigated the anti‐hypertensive effect of LMK235 in two hypertension models, angiotensin II‐infusion mice and SHRs. In this study, we first demonstrated that LMK235 attenuates the enhanced systolic BP in both animal models. Angiotensin II‐induced hypertension was effectively reduced by 7‐day daily treatment with 1 mg/kg or 3 mg/kg LMK235. However, SHRs did not exhibit a hypertension lowering effect with LMK235 at a dosage of 1 mg/kg/day (data not shown). Thus, we examined the effect of 3 mg/kg/day of LMK235 on SHRs. A BP‐lowering effect of LMK235 was observed upon injection once every 3 days rather than daily injection. In the present study, the highest suppression by LMK235 was observed 2 days after injection. On the third day, BP was restored to the level of the WKY controls. This result suggests that daily administration of LMK235 as an anti‐hypertensive medicine is not necessary and could be replaced by administration once every 2 days. In accordance with our study, other previous studies have shown that valproic acid, a class I and class IIa HDAC inhibitor, attenuated BP in SHRs or DOCA‐salt‐induced hypertensive rats.^8^, ^20^ In addition, Usui et al demonstrated that the pan‐HDAC inhibitor trichostatin A (TSA) decreased systolic BP in SHRs.^10^ However, other research groups applied HDAC inhibitors in hypertensive rats for a longer period than our group.

We examined the relationship between hypertension and the RAAS. We found that LMK235 did not affect aortic ACE1 or AT1 in either hypertension model. Hence, in this study, LMK235 was unable to suppress the expression of ACE1 and AT1. Contrary to our results, Cardinale et al showed that cardiac AT1 mRNA levels were reduced by valproic acid in SHRs.^20^

How does LMK235 reduce high BP in our experiments? One possibility may be a relaxation of vasoconstriction. We observed that LMK235 showed a higher relaxation effect in endothelium‐intact aortic rings than in endothelium‐denuded aortic rings. This result implies that endothelium cells may play a pivotal role in the relaxation from vasoconstriction rather than VSMCs. Similar to our results, it was shown that TSA treatment reversed the augmented angiotensin II‐induced contraction in the mesenteric artery of SHRs.^10^ It was also observed that LMK235 induced vascular relaxation in resistance vessels such as mesenteric arteries, which affect hypertension. Furthermore, _L_‐NAME pretreatment reduced vasorelaxation and nitric oxide production ex vivo and in vitro, indicating that vascular relaxation by LMK235 is mediated by the nitric oxide pathway.

Arterial remodelling is associated with hypertension, and it was reported that angiotensin II stimuli induces VSMC hypertrophy and hyperplasia.^11^, ^21^ In the present study, we showed that LMK235 reduces aortic wall thickness induced by angiotensin II infusion or SHRs. The anti‐hypertensive effect of LMK235 could be caused in part by suppression of VSMC proliferation. Our results showed that LMK235 resulted in decreased expression of cell cycle‐related genes, including cyclin D1 and E2F3. The p21 cell cycle arrest gene was restored in LMK235‐treated angiotensin II mice. Furthermore, the expression of calcium CaMKII α was reduced to that of the control by LMK235 administration in mice. CaMKII is required for angiotensin II‐mediated VSMC hypertrophy.^21^ Our previous study also demonstrated that CaMKIIα plays a critical role in VSMC hypertrophy and hyperplasia.^11^ Consistent with our results, the importance of CaMKIIα in pathological vascular diseases was shown in a study that used CaMKIIα inhibitor (KN 93) to inhibit bisphenol A‐induced hypertension.^22^ Among different isoforms (α, β, δ or γ) of CaMKII, we studied the role of CaMKIIα in hypertension because, only CaMKIIα responded to hypertension stimulation, such as angiotensin II.

In our previous report, we demonstrated that CaMKIIα may increase the size and number of VSMCs.^11^ In the present study, the overexpression of CaMKIIα‐induced cell cycle gene expression. CaMKIIα increased the expression of class I/IIa HDACs, except HDAC8 and HDAC9. These results suggest that HDAC1, 2, 3, 4, 5 and 7 may be associated with cell proliferation. This suggestion is consistent with one review paper, revealing that class I HDACs including HDAC1, HDAC2 and HDAC3 generally induce cell proliferation in cancer cells.^23^ According to a study published in 2016, HDAC5 expression was increased in human breast cancer, and HDAC5 was found to promote cancer cell proliferation.^24^ HDAC4 and HDAC5 have a reported similarity in amino acid sequences of 62%.^25^ CaMKII phosphorylates HDAC4 but not HDAC5. HDAC4 interacts with HDAC5, which responds to CaMKII signalling in the presence of HDAC4.^26^ In a balloon catheter injury model, CaMKIIδ2 expression was increased in rat carotid arteries, indicating that it is a positive mediator of VSMC growth. CaMKIIδ2 activates HDAC4 and HDAC5 in VSMCs.^27^ Unlike class I HDAC function in the proliferation of cancer cells, HDAC4 and HDAC5 belong to class IIa HDACs, suggesting that they play a functional role in the proliferation of blood vessels through CaMKIIδ.

LMK235 treatment significantly inhibited the expression of cell cycle genes and CaMKIIα in the aortas of angiotensin II‐infusion mice. In addition, LMK235 suppressed the CaMKIIα‐induced cell cycle genes in A10 cells. LMK235 reduced the expression of all classes of HDACs including HDAC6 in the absence of CaMKIIα. Therefore, class I HDACs may be indirectly involved in the reduction of CaMKIIα‐induced HDAC4 and HDAC5 by LMK235. LMK235 was previously reported as a class IIa preferential HDAC inhibitor.^12^ However, in our hands, LMK235 showed no inhibition of HDAC4 up to 100 μmol/L and only very modest inhibition of HDAC5 (IC_50_ around >20 μmol/L). IC_50_ values of LMK235 for HDACs newly determined in our laboratory are presented in Table 1. Inhibition of class I HDACs was similar to the results reported by Marek et al (2013). These findings on the HDAC‐inhibitory profile of LMK235 suggest that the effects observed with LMK235 in this study are most likely mediated by class I HDACs. Immunoprecipitation showed that HDAC4 and HDAC5, but not HDAC6, were associated with class I HDACs including HDAC1, HDAC2 and HDAC3 in VSMCs. Results of the immunoprecipitation experiment indicated that HDAC5 strongly interacted with HDAC1 and HDAC2, whereas, HDAC5 bound weakly to HDAC3. These findings were consistent with those of the previous study.^28^ Still, class IIa HDACs may play a significant non‐canonical role by recruiting catalytically active class I HDACs to their substrate.^29^ Thus, inhibition of class I HDACs (as performed with LMK235) and knockdown of class IIa HDACs (as performed with HDAC5) may explain the anti‐hypertensive effects observed in this study. The knockdown of HDAC5 resulted in complete suppression of CaMKIIα‐induced VSMC hyperplasia in this study, agreeing with reports of HDAC5 exerting proliferative effects.^30^

**Table 1:** IC_50_ [μmol/L] values for LMK235 and TSA

Compound	HDAC1	HDAC2	HDAC3	HDAC4	HDAC5	HDAC6	HDAC7	HDAC8	HDAC9
LMK235	0.030	0.055	0.037	>20	>20	0.050	>20	1.119	>20
TSA	0.002	0.005	0.002	3.135	ND	0.040	1.110	0.457	4.337

The in vitro inhibitory activity of compound LMK235 against each HDAC isoform was determined using the HDAC Fluorogenic Assay Kit from BPS Bioscience. The IC_50_ values were determined using 0.001, 0.003, 0.01, 0.03, 0.1, 0.3, 1, 3 and 10 μmol/L of an inhibitor. Trichostatin A (TSA) was used as the reference compound. ND indicates not determined.

In summary, the present study showed that LMK235 is a class I and HDAC6‐preferential HDAC inhibitor and attenuates hypertension through relaxation of vasoconstriction or suppression of aortic wall thickness. LMK235 or knockdown of HDAC5 inhibited angiotensin II‐induced VSMC hyperplasia. CaMKIIα is closely related to arterial growth in angiotensin II‐induced hypertension. Although the detailed mechanism of LMK235 in the regulation of vasoconstriction remains elusive, pharmacological inhibition of class I HDACs or knockdown of HDAC5 could be a novel therapeutic strategy for the treatment of hypertension.

## CONFLICT OF INTEREST

None of the authors have any conflicts of interest to declare.

## AUTHOR CONTRIBUTIONS

SYC HJK: conceptualization; SYC LJ YR YMS GRK SS: formal analysis; HJK MHJ: funding acquisition; SYC HJK: investigation; SYC LJ YR YMS GRK SS MP: methodology; TK SJK HJK MHJ: resources. HJK YMS: writing ‒ original draft. HJK TK MHJ MUK: writing ‒ review and editing.
