# Supplementary material for: Histone deacetylase inhibitor LMK235 attenuates vascular constriction and aortic remodelling in hypertension
Source: J Cell Mol Med. 2019 Feb 7;23(4):2801–12. doi: 10.1111/jcmm.14188 (PMC6433685; doi:10.1111/jcmm.14188)

HDAC Inhibitor LMK235 Attenuates Vascular Constriction and Aortic Remodeling in Hypertension

Sin Young Choi[1,2,3], Hae Jin Kee[1,2*], Simei Sun[1,2,3,4], Young Mi Seok[5], Yuhee Ryu[1,2], Gwi Ran Kim[1,2], Seung-Jung Kee[6], Marc Pflieger[7], Thomas Kurz[7], Matthias U. Kassack[7], and Myung Ho Jeong[1,2*]

[1]Heart Research Center of Chonnam National University Hospital, Gwangju 61469, Republic of Korea

[2]Hypertension Heart Failure Research Center, Chonnam National University Hospital, Gwangju 61469, Republic of Korea

[3]Molecular Medicine, Brain Korea 21 PLUS, Chonnam National University Graduate School, Gwangju 61469, Republic of Korea

[4] Zhoushan Hospital, Zhejiang University School of Medicine, No 739, Dingshen Road Lincheng New District Zhoushan Zhejiang 316021, China

[5]National Development Institute of Korean Medicine, 94 Hwarang-ro, Gyeongsan-si, Gyeongsangbuk-do, Republic of Korea

[6]Department of Laboratory Medicine, Chonnam National University, Medical School and Hospital, Gwangju 61469, Republic of Korea

[7]Institute of Pharmaceutical and Medicinal Chemistry, Heinrich Heine University Düsseldorf,

Universitätsstr. 1, 40225 Düsseldorf, Germany

1. Supplemental Table 1

Online Supplemental Table 1. Primers for reverse transcription polymerase chain reaction (RT-PCR)

Gene (rat or mouse)	Primer sequence (5′ to 3′)
HDAC4 (rat)	F: CTGACGCTGCTAGCAATGAG<br>R: TTCCAAGGGCAGTGAGAACT
HDAC5 (rat)	F: AGCACCTCTTCACCACAGGT<br>R: GTCCCATAGAGCAGGGTGTG
ACE1 (mouse)	F: CAGTGTCTACCCCCAAGCAT<br>R: TTCCATCAAAGACCCTCCAG
ACE1 (rat)	F: GTACAGAAGGGCTGGAATGC<br>R: CGTGCACTGCTTAATCCTGA
AT1 (mouse=rat)	F: GGAAACAGCTTGGTGGTGAT<br>R: GGCCGAAGCGATCTTACATA
Cyclin D1 (mouse)	F: AACTACCTGGACCGCTTCCT<br>R: CCACTTGAGCTTGTTCACCA
Cyclin D1 (rat)	F: CTGCATGTTCGTGGCCTCTA<br>R: CCTCTGGCATTTTGGAGAGG
18S rRNA (mouse)	F: GTGGAGCGATTTGTCTGGTT<br>R: CGCTGAGCCAGTCAGTGTAG
18S rRNA (rat)	F: CATTCGAACGTCTGCCCTAT<br>R: GCCTTCCTTGGATGTGGTAG

Gene<br><br>(rat or mouse)	Primer sequence<br><br>(5′ to 3′)
E2F3 (mouse)	F: ATCCAAAGCTGTACCCTGGA<br><br>R: TGGGTACTTGCCAAATGGAT
E2F3 (rat)	F: ACACCACGCCACAAGGACCC<br><br>R: TCTTCCTTTGCCCTTGGGGG
p21 (mouse)	F: CGGTGGAACTTTGACTTCGT<br><br>R: GAGTGCAAGACAGCGACAAG
p21 (rat)	F: AGTTAAGGGGAATTGGAGGCAGGC<br><br>R: CTCAGGTAGATCTTGGGCAGCCCT
CaMKIIα<br><br>*(mouse=rat)*	F: ACAGAGCAGCTGATCGAAGC<br><br>R: AGGTGGATGTGAGGGTTCAG
GAPDH (mouse)	F: GCATGGCCTTCCGTGTTCCT<br><br>R: CCCTGTTGCTGTAGCCGTATTCAT
GAPDH (rat)	F: AACCCATCACCATCTTCCAGGAGC<br><br>R: ATGGACTGTGGTCATGAGCCCTTC

F, forward; R, reverse.

Gene<br><br>(rat or mouse)	Primer sequence<br><br>(5′ to 3′)
HDAC1 (rat)	F: AGGGCACCAAGAGGAAAGTCTGTT<br><br>R: TTCAGACTTCTTCGCATGGTGCAG
HDAC2 (rat)	F: CAGTTGCCCTTGATTGTGAA<br><br>R: AGCTTGCATTTGAACACCAG
HDAC3 (rat)	F: CAAGACCGTGGCGTATTTCTACGA<br><br>R: GCCCAGTTGATGGCAATATCACAG
HDAC7 (rat)	F: AAAGACAAGAGCAAGCGAAGTGCC<br><br>R: CCTCTCCAGGGATTTCTTGGGTTT
HDAC8 (rat)	F: AATGTTCCTAATCTGGATGGGCCC<br><br>R: CTTCACAAGGGAATCGCAGATGCT
HDAC9 (rat)	F: CCCACCACACATCACTGGATCAAA<br><br>R: CATTGTTTGGTGAACTGGGACCTG

2. Supplemental Figures Legends

Online Figure 1. Effect of CaMKII α transfection on cell proliferation in A10 cells.

(**A–B**) A10 cells were transiently transfected with the indicated concentrations (ng) of pCMV-SPORT6-CaMKIIα for 24 h (A) or 48 h (B). MTT assay was performed.

Online Figure 2. Effect of CaMKII α transfection on cell-cycle gene expressions in A10 cells.

(**A–B**) A10 cells were transiently transfected with the indicated concentrations of pCMV-SPORT6-CaMKIIα for 24 h. Transcript levels for CaMKIIα and cyclin D1 were examined by qRT-PCR. Target genes were normalized to 18S rRNA. Data are expressed as mean ± SE of three independent experiments.

Online Figure 3. Effect of LMK235 on cell viability in A10 cells.

After serum starvation, A10 cells were treated with the indicated concentrations of LMK235 for 24 h. MTT assay was performed. Data are expressed as mean ± SE of three independent experiments.

Online Figure 4. Effect of CaMKII α transfection on expression of class I and IIa HDACs in A10 cells.

(**A–B**) A10 cells were transiently transfected with the indicated concentrations of pCMV-SPORT6-CaMKIIα for 24 h. Transcript levels of class I (HDAC1, 2, 3, and 8) and class IIa (HDAC4, 5, 7, and 9) were analyzed by qRT-PCR. Data are expressed as mean ± SE of three independent experiments. $^{*}P < 0.05$, $^{**}P < 0.01$, and $^{***}P < 0.001$ versus empty vector; NS indicates not significant.

Online Figure 5. LMK235 reduces the protein levels of HDAC1 and HDAC2 in CaMKII α transfected A10 cells.

A10 cells were transiently transfected with pCMV-SPORT6-CaMKIIα. On the next day, A10 cells were treated with LMK235 for 18 h. The protein levels of HDAC1 and HDAC2 were evaluated using densitometry. $^{###}P < 0.001$ versus CaMKIIα transfection group; NS indicates not significant.

Online Figure 6. LMK235 reduces the protein levels in class I and IIa/b HDACs in A10 cells.

A10 cells were serum starved for 3 h and treated with LMK235, or vehicle, for 18 h. (A) Protein images of class I HDACs (HDAC1, HDAC2, and HDAC3), class IIa HDACs (HDAC4 and HDAC5), and class IIb HDAC (HDAC6) in A10 cells. (B) HDAC proteins were quantified using densitometry. $^{***}P < 0.001$ versus vehicle group.

Supplementary Fig. 1

A

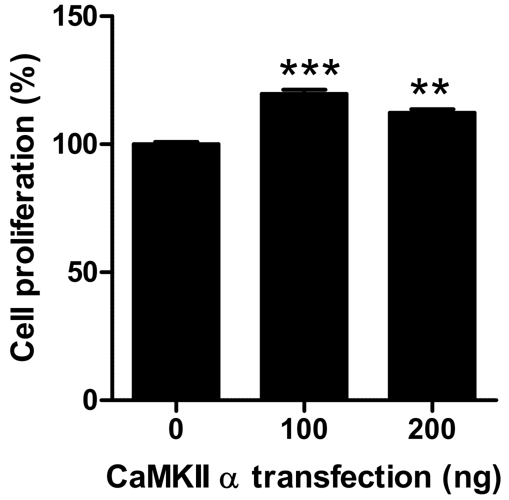

B

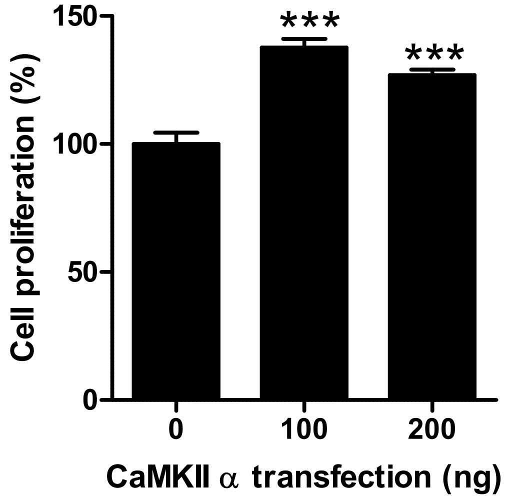

Supplementary Fig. 2

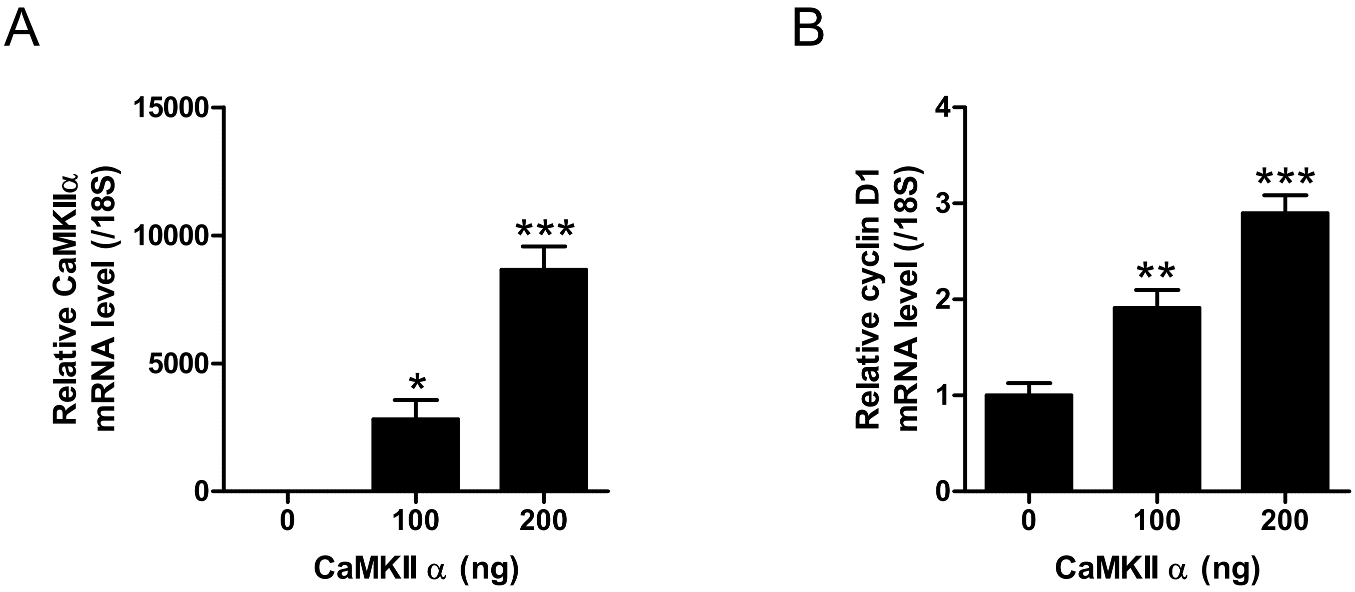

Supplementary Fig. 3

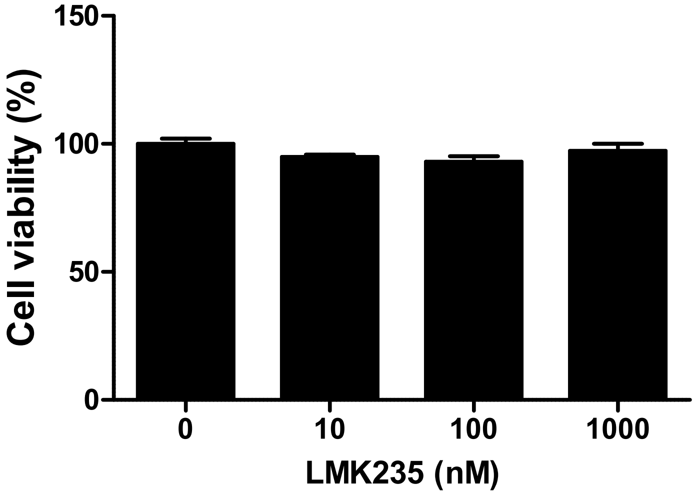

Supplementary Fig. 4

A

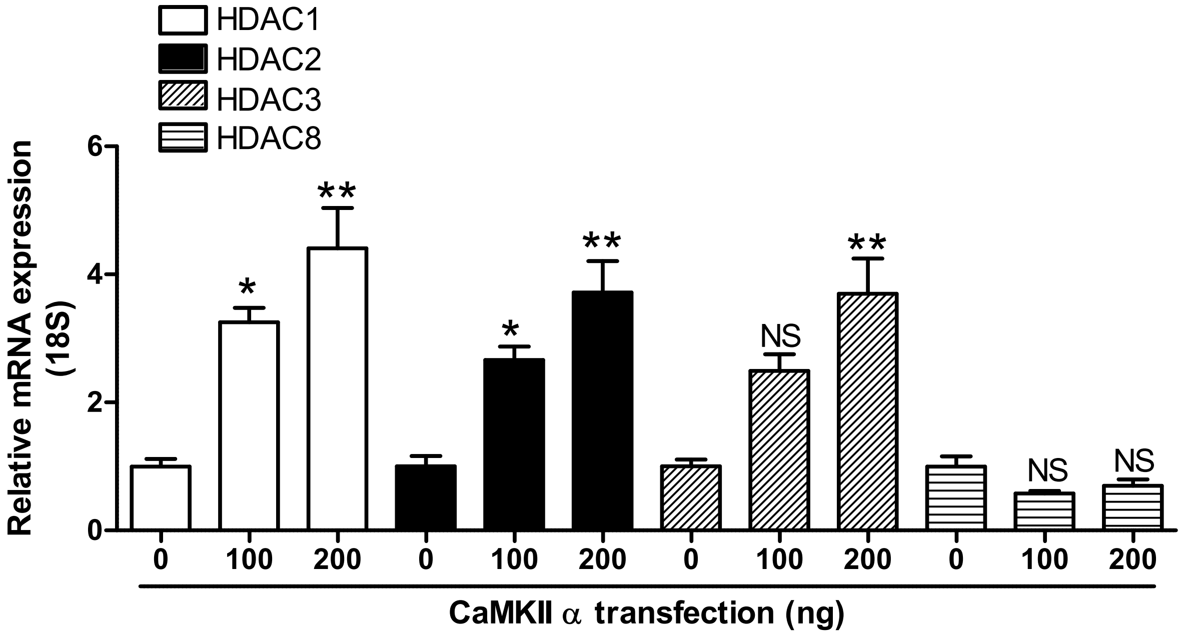

B

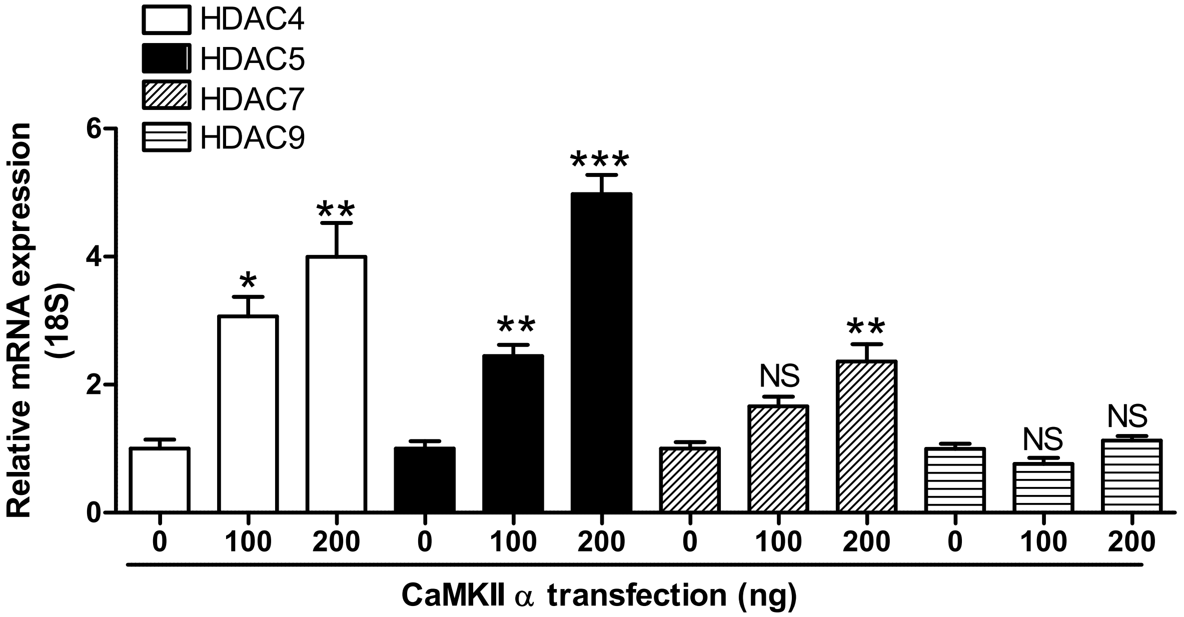

Supplementary Fig. 5

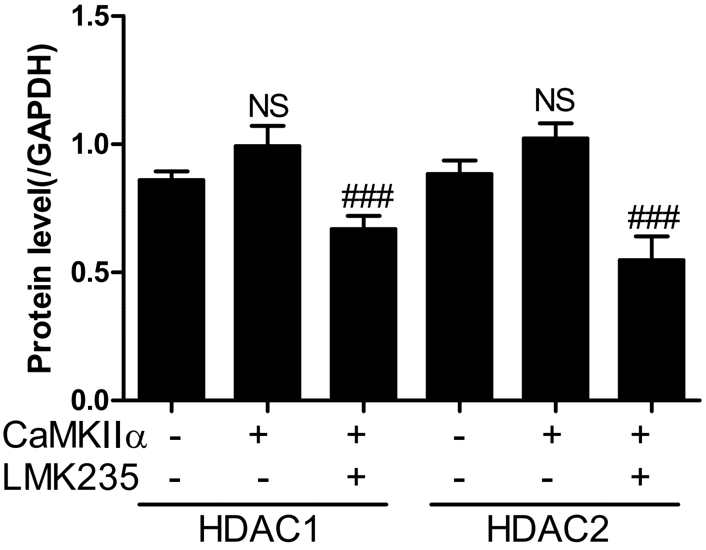

Supplementary Fig. 6

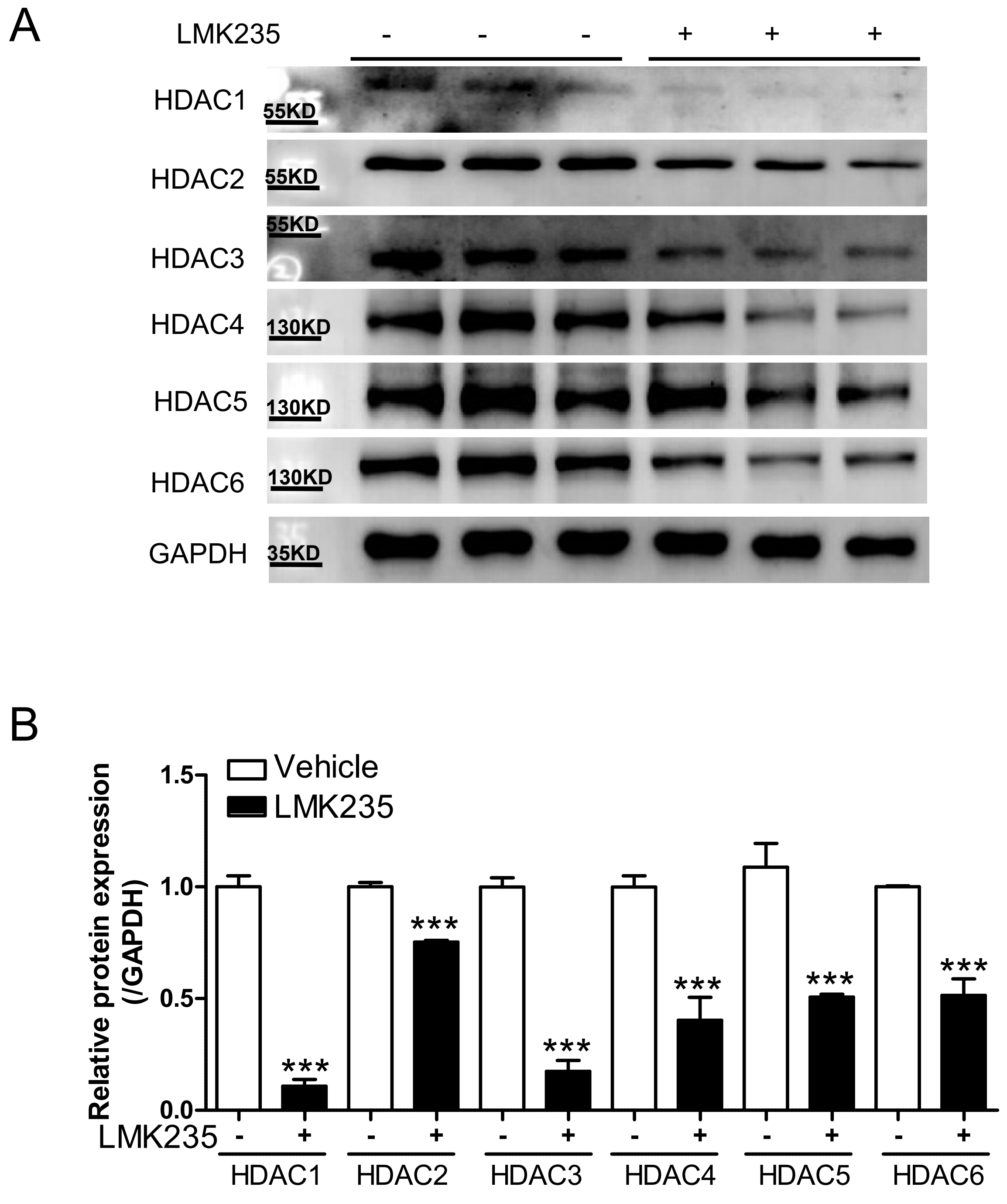

Supplement: Supplementary file 1 [file JCMM-23-2801-s001.pdf]
